# Home-based physical exercice with additional cognitive training for improving mobility in older adults: a secondary analysis of the COVEPIC randomized controlled trial

**DOI:** 10.1186/s11556-026-00415-z

**Published:** 2026-05-06

**Authors:** Emma Gabrielle Dupuy, Caroll-Ann Blanchette, Florent Besnier, Christine Gagnon, Thomas Vincent, Tudor Vrinceanu, Juliana Breton, Kathia Saillant, Josep Iglesies-Grau, Sylvie Belleville, Martin Juneau, Paolo Vitali, Anil Nigam, Mathieu Gayda, Louis Bherer

**Affiliations:** 1 Research center and Centre ÉPIC, Montreal Heart Institute, Montreal, Canada; 2 INSERM, CAPS UMR 1093, Université Bourgogne Europe, Dijon, France; 3 Department of Medicine, Faculty of Medicine, University of Montreal, Montreal, Canada; 4 Department of Psychology, University of Quebec in Montreal, Montreal, Canada; 5 Research center, Institut Universitaire de Gériatrie de Montréal, Montreal, Canada; 6 Department of Psychology, University of Montreal, Montreal, Canada; 7 McGill Research Centre for Studies in Aging, Douglas Mental Health University Institute, Montreal, Canada; 8 Department of Neurology and Neurosurgery, McGill University, Montreal, Canada

**Keywords:** Aging, Gait Speed, Balance, Cognition, Physical Exercise, Remote Monitoring, Multidomain intervention

## Abstract

**Background:**

Home-based physical exercise is an accessible strategy to help maintain physical functioning in adults over 50, but mobility gains may be limited without direct supervision. By improving cognitive processes involved in motor control, cognitive training may help enhance the effectiveness of home-based physical exercise in preventing age-related decline in mobility. This study compares the effects of a six-month home-based physical exercise, with or without cognitive training, on gait speed and balance.

**Methods:**

127 community-dwelling adults aged 50–87 years (mean 65.20 ± 7.93; 76% women) were randomly assigned to (1) home-based, remotely monitored physical exercise alone (*n* = 64; 72% women) or (2) combined with cognitive training (*n* = 63; 81% women) for six months. The primary outcome was the change from baseline in usual gait speed, measured by the 4-meter walking test. Secondary outcomes included fast gait speed, balance (one-leg stance test), and lower-limb strength (five-time sit-to-stand test), all of which were assessed via videoconference at 3 and 6 months. Changes in outcomes were analyzed using adjusted mixed linear models with intervention group, time (ΔT0–T3, ΔT0–T6), and their interaction as fixed effects.

**Results:**

Change in usual gait speed did not differ between groups. In contrast, the combined group showing greater improvements than the physical exercise alone group in fast gait speed (+ 0.07 m/s; F(1,199) = 6.24, *p* = 0.013, η^2^p ≃0.03) and one-leg balance test (+ 3.83 s; F(1,202) = 6.05, *p* = 0.015, η^2^p ≃0.03) in response to intervention. No significant group × time interaction was observed, indicating similar changes at 3 and 6 months.

**Conclusion:**

Adding cognitive training to home-based physical exercise may improve fast gait speed and balance in adults aged 50 and above. These findings support the interplay between sensorimotor and cognitive functions in older adults and suggest that combined physical and cognitive interventions could help prevent age-related mobility decline.

**Clinical Trial Registration ID:**

COVEPIC was retrospectively registered on November 19, 2020. Clinical trial Identifier: NCT04635462. https://clinicaltrials.gov/ct2/show/record/NCT04635462?term=NCT04635462&draw=2&rank=1

**Supplementary Information:**

The online version contains supplementary material available at 10.1186/s11556-026-00415-z.

## Introduction

Currently, people who reach the age of 60 can expect to live an average of 20 years more; a trend that is expected to continue over the next two decades, with the number of people aged 85 and older projected to triple [1]. However, living at an ‘oldest-old’ age (≥ 85 years) remains a collective blind spot, as this gain in longevity is not mirrored by the years lived without disability. One possible explanation lies in the fact that even high-quality and accessible healthcare systems in industrialized countries may still struggle to deliver effective, tailored strategies to prevent age-related functional decline [2].

Mobility is a key factor in maintaining functional independence in older adults [3]. Usual gait speed is a clinically meaningful mobility marker, considered a functional vital sign and associated with health outcomes and mortality in this population [4, 5]. Older adults walking at speeds below 0.8 m/s face an increased risk of loss of autonomy and mortality [6], while an improvement of 0.1 m/s or more in gait speed is linked to meaningful gains in activities of daily living [7]. Although seemingly simple, walking depends on complex motor, neuromuscular, cardiorespiratory, and musculoskeletal functions, all known to be affected by biological aging [8]. As a result, mobility restrictions increase with age, impairing movement safety, independent daily functioning, and increasing the risk of falls [3, 9].

Maintaining regular physical activity, including aerobic exercise and multicomponent training (strength, balance, and flexibility exercises), remains the primary recommendation to prevent age-related functional limitations and to preserve physical functions [10, 11]. Remote monitoring by a health professional has been shown to successfully engage inactive individuals while enhancing long-term retention of physical exercise routines [12, 13], but the absence of direct supervision and feedback on physical exercises may reduce the benefits for physical functions affected by aging, particularly lower-limb functions supporting mobility [14]. In response, multidomain interventions adding cognitive training to home-based physical exercise benefits may offer a relevant approach to address this limit and improve gains in mobility. Several studies suggest that attention-executive computerized training, involving repeated practice of visuomotor tasks targeting a specific cognitive ability (i.e., processing speed, executive function; here implemented as a dual-task), may improve physical functions in older adults, including functional mobility markers such as usual gait speed [15, 16].

The sequential combination of cognitive training and physical exercise within a single intervention may simultaneously target multiple components of individual functioning that decline with advancing age, thereby improving functional gains [17, 18]. Contemporary approaches posit that the cardiometabolic effects of physical exercise, and their impact on brain functioning, facilitate the neurocognitive plasticity induced by cognitive training [17, 18]. This synergy may increase improvements in cognitive functioning, particularly in executive functions, which rely heavily on the integrity of the prefrontal cortex, a richly vascularized region that is especially vulnerable to age-related decline [17, 18]. The added value of combining cognitive and physical exercise for older adults’ executive functions, compared with physical exercise alone, was confirmed in the main results of this trial [19]. From this perspective, multidomain interventions are grounded in the idea that physical exercise creates a neurobiological environment conducive to the effectiveness of cognitive training, yielding broader and more robust effects than either modality alone [18, 20].

One plausible mechanism underlying the benefits of computerized cognitive training in older adults’ physical function is the age-induced permeation of sensorimotor functioning with cognition [21]. With aging, mobility tends to rely on the engagement of overlapping neural resources supporting executive and motor control, such as the prefrontal cortex, basal ganglia, and cerebellum [22, 23]. This overlap is likely enhanced by compensatory recruitment of prefrontal areas due to age-related declines in neurophysiological structures that support gross motor skills [24]. Evidence of such far-reaching transfer effects of cognitive training interventions remains scarce and sometimes contested [25, 26], and previous research has not reached a consensus regarding the added value of these interventions compared to physical exercise alone for mobility, especially when exercise is performed by older adults from home in autonomy [27].

The COVEPIC study was conducted during the COVID-19 pandemic to address the dual constraints of social isolation and limited access to sports facilities, while investigating relevant research questions regarding the prevention of age-related functional decline [19, 28]. The main results of the COVEPIC randomized controlled trial showed that combining cognitive training with physical exercise led to greater improvements in executive function [19]. In this secondary analysis, we aimed to explore whether cognitive training enhances mobility outcomes when combined with physical exercise in a home-based, remotely monitored intervention, drawing on the trial’s physical function assessments. Our main hypothesis was that combining home-based cognitive training with physical exercise would improve usual gait speed to a greater extent than physical exercise alone. Secondarily, we hypothesized that a greater benefit of the combined intervention would also be observed in other physical outcomes, assessing balance, lower limb strength, and fast gait speed.

## Methods

### Design

This study is a prespecified secondary outcome analysis of the COVEPIC trial, a randomized, single-blinded trial with two parallel arms investigating the effects of home-based physical exercise and cognitive training in community-dwelling adults aged 50 and older [28]. The trial was conducted during the COVID-19 pandemic, from May 2020 to October 2021. Details of the trial design, recruitment, interventions, and participants’ follow-up have been described previously25 (doi: 10.1186/s13063-021-05476-2) after registration on ClinicalTrials.gov, the NIH public database platform (NCT04635462). The primary findings regarding changes in cognitive functioning (executive function, processing speed, and verbal memory) have also ben published [19]. The Montreal Heart Institute’s Research Ethics Board (FWA00003235) approved this research project (MHI 2019–2785), and all participants gave their written informed consent before participating.

### Participants

Research participants were recruited from active members of the Montreal Heart Institute’s EPIC center and the community using advertising and online study announcements (e.g., videos, interviews, newsletters). Eligible individuals had to be aged 50 and older and have access to a computer or a tablet (i.e., iPad or Android) connected to the Internet. Individuals were excluded if they had any significant cognitive impairment (score lower than 19/23 on the telephone version of the Mini-Mental State Examination, t-MMSE) [29], non-cardiopulmonary limitation to physical exercise (e.g., arthritis), or severe physical exercise intolerance, respiratory or cardiovascular disease (e.g., severe asthma, COPD, severe COVID-19-related symptoms, chronic heart failure, somatic aortic stenosis, atrial fibrillation, malignant arrhythmias, or documented atherosclerosis disease). Participant selection was made by phone. During this call, the research member checked if the individual had access to the Internet and computerized technologies and did not present any major physical or medical limitations to physical exercise. Contact information was collected, and a consent form was emailed. During a second call, the t-MMSE and the Physical Activity Readiness Questionnaire for Everyone (PAR-Q+) were administered to individuals who consented to participate in the COVEPIC study. At the same time, participants completed a medical questionnaire, which a research team member reviewed to confirm their eligibility. When the participant’s medical condition was unclear, a physician validated their eligibility.

### Randomization and blinding

A 1:1 ratio allocated participants to one of the two study arms: 1) home-based, remotely monitored physical exercise alone or 2) home-based, remotely monitored physical exercise combined with cognitive training. The sequence was computer-generated by the Montreal Health Innovations Coordinating Center (an organization independent of the research team). A staff member not involved in any procedure or analysis for this trial performed and communicated the randomization to the research staff, who took charge of training follow-up of the participants. The assessors were blinded to the group allocation, and participants were asked not to share any information about their training during assessment sessions.

### Intervention procedure

The intervention lasted six months, during which participants were encouraged to engage in regular physical exercise with or without cognitive training, depending on their assigned study group. To complete their training, they received a document of guidelines and an introductory phone call from their training coach to orient them on the nature, intensity, and frequency of their physical exercise and cognitive training sessions. During the intervention, for each cognitive or physical exercise session, participants had to report the following in a training agenda: the type of activity (physical or cognitive exercise for the combined group), its nature (e.g., muscle training, running), duration, and their perceived exertion during exercise, rated on a 10-point Borg Scale. The intervention combined recommendations individualized weekly follow‑up, and access to instructional materials, without following a specific standardized program or strict prescription. Participants were allowed to maintain or modify their usual exercise habits and to choose the activities they preferred. Once a week, the coach conducted a follow‑up call to review the agenda, provide tailored advice, and support adherence.

Recommendations for physical exercise included performing at least five 15-minute sessions each week. Online exercise video capsules were made available to participants via Facebook or YouTube (https://tinyurl.com/epicicm) and could be combined with Zoom training sessions and outdoor physical exercise. Several activities, including aerobic and resistance training, as well as yoga and stretching, were proposed in the video capsules developed by the kinesiologists at the Montreal Heart Institute’s EPIC prevention center. Participants were free to engage in various activities (e.g., aerobic, resistance, mind–body, stretching), and these activities were classified after data collection. Each physical exercise session reported by participants in their agenda was quoted according to the Physical Activities categories of the Compendium of Physical Activities 28 (https://pacompendium.com/), as previously described in the clinical trial [19]. The metabolic energy expenditure was then calculated per week (MET-Min-Week) and averaged between intervention week (from T0 to T6) to obtain the mean weekly exercise dose overall and according to six main categories: aerobic (e.g., running, biking), resistance, mind-body (e.g., yoga, tai-chi), mobility and balance, stretching, activity mixed with aerobic (e.g., combining resistance and aerobic).

Participants enrolled in the combined group were encouraged to add three 30-minute sessions of cognitive training per week. The cognitive training proposed combined process- and strategy-based approaches [30], and provided training developed by our team to train older adults [31]. The three sessions included two computerized cognitive task sessions and one video capsule, inspired by the MEMO+ program [32]. The computerized training included three visual discrimination tasks: working memory training (i.e., N-Back task [33]), dual-task training (i.e., Dual-task [31]), and inhibition training (i.e., modified Stroop task [34]). Each task lasted approximately 15 min and consisted of its own set of visual stimuli and corresponding button symbols. Participants were required to complete the tasks as quickly as possible while maintaining accuracy. Participants received adaptive feedback, and the difficulty level gradually increased throughout their training. An educational video program proposed capsules dedicated to developing strategies to manage multiple aspects of their daily life, such as memory, anxiety, nutrition, and sleep. Memory capsules instructed participants about age-related changes in memory and taught mnemotechnics (e.g., face-name association, visual imagery).

### Outcomes

Participants’ profiles in terms of global cognitive functioning, physical fitness, and physical activity level were assessed at baseline using their scores in the remote version of the Montreal Cognitive Assessment (MoCA [35]), the VO_2_ max estimation of Matthew’s questionnaire [36], and the Physical Activity Scale for Elderly (PASE [37]). At baseline, sex, age, anthropometric data (height and body mass), medical conditions, and lifestyle habits were also collected using a self-reported questionnaire.

#### Physical functions assessment

Physical function assessment included balance, functional mobility, and lower limb muscle strength. The selected tests were validated for use with older adults and adapted for remote administration via videoconference supervision. Testing sessions assessing physical performance lasted 20 to 30 min and were performed at baseline and after three and six months of intervention.

To ensure an appropriate setup from participants’ homes, a demonstration video of the tests and guidelines for functional testing, describing the space and materials required, were sent two days before the session. Participants were first asked to wear stable, flat shoes and to report any recent pain, limitations, or health changes. The assessor then verified the testing environment with the participant, including the exact 4‑meter walking distance (measured with a tape measure and delimited by pre‑marked floor landmarks), the use of a stable standard-height chair, and the absence of obstacles on the track. During the testing session, the assessor maintained continuous visual monitoring via videoconference, provided real‑time corrections (e.g., foot placement, starting position), and ensured that all safety criteria were met before each test. All these dispositions converge with the recommendations published for remote gait and balance assessment [38]. Details of the remote functional tests modality are also available in the published protocol [28].

Balance was assessed using the 60-second one-leg balance test [39]. Participants had to stay on one leg as long as they could with their eyes open and no assistance. The test began when the participant’s foot left the ground and ended if it touched the ground, with a maximum duration of 60 s. Two trials were performed for each leg, and the best duration on the dominant leg was kept. Functional lower limb strength was assessed by the Five-Time-Sit-to-Stand test [40]. For this test, participants had to transfer from a seated to a standing position and back to a seated position as quickly as possible, without using their arms as leverage. Participants began the tests seated in the middle of the chair. They were to start at the assessor’s go and perform the movement without resting their back or leg on the chair between repetitions. The test was performed twice, and the results are presented as the average of the two durations. For functional mobility, gait speed was assessed at the usual and fast pace on a 4-meter track [41]. Two landmarks were positioned 4 m apart to offer an unobstructed straight line. The participant performed a dynamic start, i.e., started with their toes behind the starting line and had to walk “usual speed, just as if [they] were walking down the street to go to the store.” (Usual-pace gait speed) or “to walk as fast as they can, but without running” (Fast-paced gait speed). Gait speed was measured using a stopwatch over the 4-meter track. The three trials were performed at usual and fast paces, with 20 to 30 s of rest between each trial. The results are presented as the average duration for both usual gait speed and fast gait speed.

Outcomes were defined as the changes in physical functions, calculated by subtracting baseline measurements from those at three and six months for each variable: Δ usual gait speed, Δ fast gait speed, Δ five-time sit-to-stand, and Δ one-leg balance test. Considering the clinical relevance and validity of usual gait speed in relation to health and functional status among older adults [7, 42], Δ usual gait speed was selected as the primary indicator of intervention efficacy. To interpret the clinical significance of observed changes, Minimal Clinically Important Differences (MCID) reported in the literature will be considered: 0.05 m/s for usual gait speed and fast gait speed, and 0.1 m/s represents a robust clinical change, 2–3 s for the five-time-sit-to-stand, and approximately 5 s for the one-leg balance test [7, 40, 43].

### Sample sizing

Sample size estimation was performed a priori for the primary outcome of the COVEPIC randomized controlled trial, namely executive function, as defined in the original study protocol. Using reference values from the literature for physical exercise training alone [44], and assuming a mean of 0.239 for the physical training group and 0.425 for the combined training group (mean difference = -0.186, SD = 0.298), a sample size of 49 participants per group was required to achieve 80% power to detect an effect size of 0.624, using a two-sided two-sample t-test (α = 0.05). The physical function outcomes analyzed in the present manuscript are secondary outcomes of the trial, for which no specific power calculation was planned.

### Statistical analysis

Analyses were performed using SPSS statistical software, version 29.0 (SPSS Inc., Chicago, IL), with a significance level set at 0.05. Baseline and training characteristics were presented as means ± standard deviations, or frequencies (percentages). Between-group differences in characteristics and physical exercise practice were examined using t-tests for continuous variables and Chi-squared tests for nominal/ordinal variables. Before the analyses, assumptions underlying the models were checked. Outliers on outcome were identified using Tukey’s rule, defined as values beyond 1.5 times the interquartile range from the first or third quartile, and were excluded accordingly.

Changes in physical functions were compared across groups using mixed linear model analyses, with the *intervention group* (combined, physical exercise), *follow-up times* (Δ_T0−T3_, Δ_T0−T6_), and their *interaction* (group × time) included as fixed effects. Estimates were adjusted for sex, age, mean weekly physical exercise dose (MET-Min-Week) over the 6 months of intervention, and baseline performance on the outcome of interest. Models included random intercepts to account for inter-individual variability.

Mixed linear models were estimated using restricted maximum likelihood (REML). Several covariance structures were tested, and the variance component’s structure was retained for the final analysis, as it provided a better fit than more complex structures that incorporated correlations between measures. Bonferroni corrections were applied to all post hoc pairwise comparisons.

## Results

### Participants’ characteristics

**Fig. 1:**
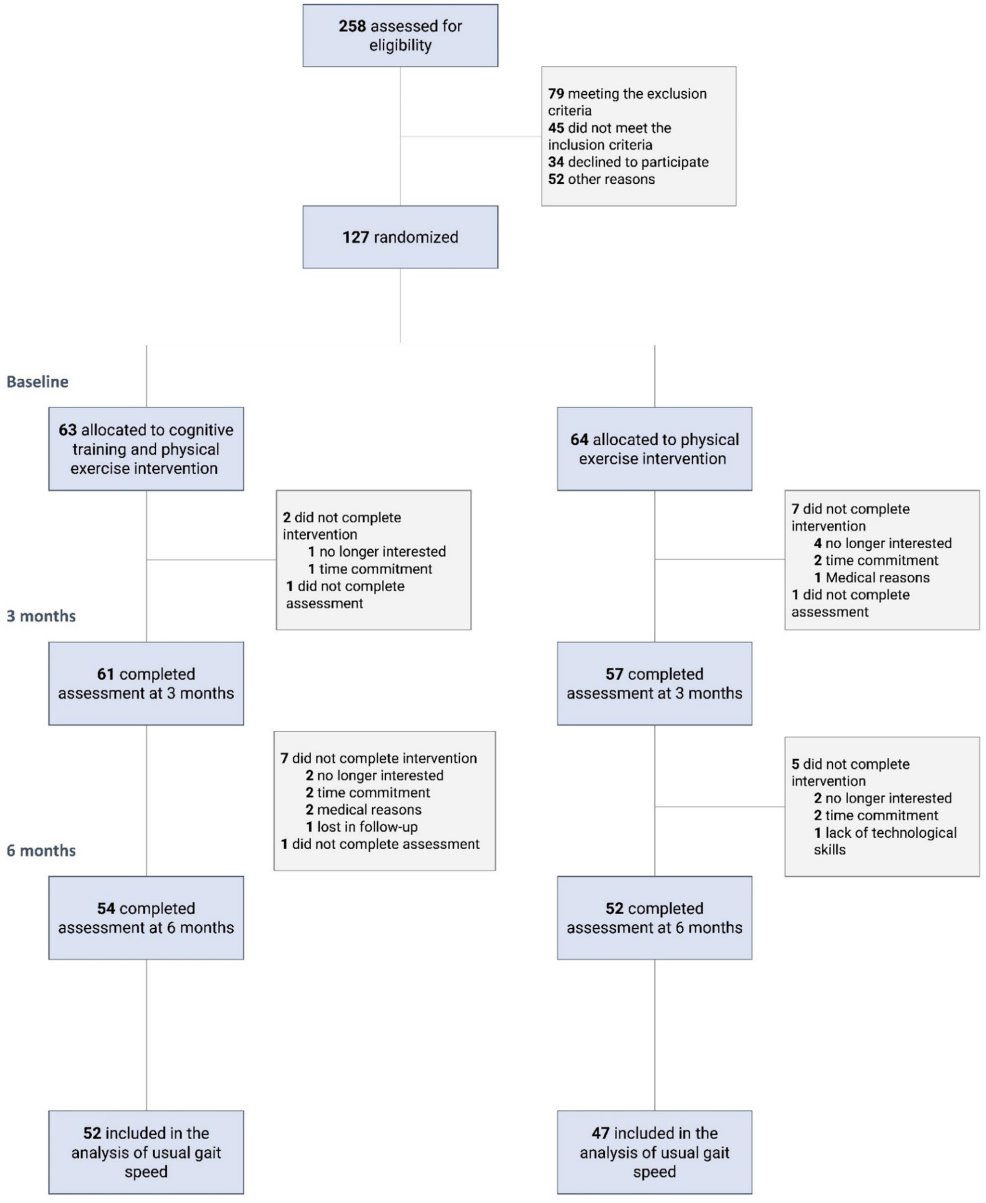
COVEPIC flowchart on primary outcome

Figure 1 provides an overview of participant flow. A total of 258 participants were called for eligibility screening. One hundred twenty-seven participants were enrolled in the study, with 64 randomized to the physical exercise group and 63 to the combined group (physical exercise plus cognitive training). 76% of participants were women, with no significant differences in demographic and clinical characteristics between the two intervention groups at baseline (Table 1). On average, participants were aged 65.20 ± 7.93 years (range 50–87), with 50% of individuals aged 65 years or older, and walked at a usual pace of 1.03 m/s at baseline.

**Table 1:** Baseline and training characteristics of the participants according to their study group

Variables	Cognitive training and physical exercise	Physical exercise	*P*-value*
	*N* = 63	*N* = 64	
Baseline characteristics			
Age (years)	64.51 (8.22)	65.88 (7.67)	0.33
Sex (% of female)	80.95	71.88	0.36
Height (m)	1.66 (0.1)	1.64 (0.1)	0.17
Body mass (kg)	76.49 (17.1)	71.58 (14.5)	0.09
BMI (kg/m^2^)	27.80 (5.37)	26.71 (4.80)	0.24
Education (years)	17.01 (2.73)	17.03 (2.70)	0.96
MoCA (0–30 score)	27.38 (2.02)	27.00 (2.34)	0.33
Phonologic verbal fluence (number of words in 60s)	14.75 (4.04)	15.89 (3.63)	0.10
MAT part C (number of switches in 30s)	27.46 (6.02)	26.36 (6.44)	0.36
GDS (0–30 score)	6.87 (5.3)	7.48 (5.9)	0.55
Usual gait speed (m/s)	1.02 (0.20)	1.04 (0.22)	0.68
Fast gait speed (m/s)	1.42 (0.29)	1.40 (0.34)	0.81
5x Sit-to-Stand (s)	10.69 (2.80)	10.40 (2.71)	0.55
One leg balance (s)	49.27 (17.66)	50.24 (16.86)	0.75
Estimated VO2 max^a^ (ml/min/kg)	25.17 (5.78)	24.08 (6.86)	0.35
PASE (0-793 Score)	127.80 (49.77)	131.14 (52.98)	0.72
Characteristics of intervention over the 6 months*			
Weekly dose of physical exercise (MET-min)	1253.78 (635.1)	1223.65 (591.6)	0.80
Weekly duration over the 6 months			
Overall intervention^b^ (min)	311.21 (149.1)	285.24 (140.8)	0.28
Overall physical exercise (min)	286.99 (148.4)	285.24 (144.4)	0.95
Aerobic exercise (min)	237.27 (143.5)	221.94 (135.3)	0.42
Resistance exercise (min)	30.74 (40.0)	41.46 (60.0)	0.29
Cognitive training (min)	29.20 (19.6)		
Computerized task (min)	17.51 (12.3)		
MEMO+ video capsules (min)	11.80 (9.2)		

Continuous variables are presented as mean (SD); categorical variables are presented as percentages

*Abbreviation*: *BMI* Body Mass Index, *GDS* Geriatric Depressive Scale, *MAT* Mental Alternation Test, *MoCA* Montreal Cognitive Assessment, *PASE* Physical Activity Scale for the Elderly

^a^ VO2 max was estimated using the Matthew questionnaire

^b^ Intervention: the combination of physical exercise and cognitive training

*Training data are reported for participants who completed the usual gait speed assessment at six months

Participants engaged in physical exercise for an average of 282 ± 144 min per week over the six months of intervention, with a rated perceived exertion of 2.93 ± 1.33 per physical exercise session on a 10-point Borg Scale, corresponding to moderate exertion. No difference was observed between the two groups. Most physical exercise sessions were dedicated to aerobic and resistance training, with each accounting for 82% and 10% of the reported exercises, respectively. Stretching, mind-body, mobility, and balance exercises were also reported, but represented only 1% to 2% of the total physical exercise volume. Finally, participants in the combined group performed an average of 29.20 ± 19.6 min per week of cognitive training over the six months, comprising 60% computerized cognitive tasks and 40% educational video capsules. The demographic and training characteristics of participants by study group are detailed in Table 1.

### Between-group comparison of change in usual gait speed

The mixed linear model did not show a significant between-group difference for the change in usual gait speed after the intervention. However, an overall increase of 0.078 m/s in usual gait speed was observed across all participants, regardless of group (95% CI [0.052, 0.104]). No effect of *follow-up time (i.e.*,* 3 or 6 months) on usual gait speed change was observed*. By the end of the 6-month intervention, 45.6% of participants achieved a robust, clinically meaningful improvement in usual gait speed (> 0.1 m/s).

#### Between-group comparison of change in other physical performances

Mixed linear model analyses of secondary outcomes revealed significant between-group differences in changes in fast gait speed and one-leg balance test duration following the intervention. Compared to the physical exercise-only group, the combined group showed a greater mean change in fast gait speed (+ 0.07 m/s, 95% CI [0.022, 0.142]; F(1,199) = 6.240, *p* = 0.013, η^2^p ≃0.03), and a greater change in one-leg balance test duration (+ 3.83 s, 95% CI [0.759, 6.894]; F(1,202) = 6.051, *p* = 0.015, η^2^p ≃0.03).

The mean change in fast gait speed for the combined group was 0.151 m/s (95% CI [0.104, 0.185]), which was almost twice the improvement recorded in the physical exercise-only group (0.078 m/s, 95% CI [0.019, 0.106]). In the one-leg balance test, the physical exercise-only group showed a mean decrease of 2.50 s (95% CI: [-5.020, -0.021]), whereas the combined group showed no significant change in one-leg stance duration. After 6 months of intervention, a robust and clinically meaningful improvement in fast gait speed (> 0.1 m/s) occurred in 60.8% of participants in the combined group and in 53.2% of the physical exercise-only group. For the one-leg balance test, 15.7% of the combined group achieved an improvement greater than 5 s, compared to 6.4% in the exercise-only group.

No significant between-group difference was found for changes in the five-time sit-to-stand. However, both groups showed a mean reduction of -0.55 s (95% CI [-0.797, -0.306]) in time to complete the test. This change did not reach the threshold for clinical significance in most participants, with only 2% showing an improvement of more than 3 s after 6 months. No significant effect of follow-up time was found for any outcome. Between-group comparisons of changes in all physical function outcomes are presented in Table 2, and graphical representations are provided in the supplementary material.

**Table 2:** Between‑group differences in change scores of physical function outcomes (ΔT0–T3 and ΔT0–T6) with 95% confidence interval

Outcome	Group	ΔT0–T3			ΔT0–T6			P-value *group*	P-value *group x time*
		N	Estimate	CI 95%	N	Estimate	CI 95%		
Usual gait speed (m/s)	Exercise	51	0.08	0.04 to 0.12	47	0.06	0.02 to 0.10	0.564	0.190
	Combined	58	0.07	0.03 to 0.11	51	0.10	0.06 to 0.14		
Fast gait speed (m/s)	Exercise	52	0.08	0.03 to 0.14	47	0.07	0.01 to 0.13	0.013	0.425
	Combined	58	0.13	0.08 to 0.19	50	0.17	0.11 to 0.23		
One-leg balance test (s)	Exercise	53	-2.51	-4.76 to 1.28	47	-3.44	-6.51 to -0.09	0.015	0.696
	Combined	59	2.77	-0.18 to 5.56	51	-0.75	-3-16 to 3.01		
Five-time sit-to-stand (s)	Exercise	52	-0.26	-0.68 to 0.17	45	-0.80	-1.26 to -0.34	0.426	0.187
	Combined	58	-0.72	-1.13 to -0.32	50	-0.69	-1.12 to -0.25		

Estimate: Estimated marginal mean (adjusted for sex, age, mean weekly dose of physical exercise over the 6 months (in MET)) from the mixed linear model for the change from baseline to 3 months (ΔT0–T3) and 6 months (ΔT0–T6)

*CI 95%* Confidence interval at 95%

## Discussion

This study examined changes in physical function among adults aged 50 and older following six months of remotely monitored home-based physical exercise, with or without cognitive training. Contrary to our primary hypothesis, the combined intervention did not result in greater improvements in usual gait speed compared to physical exercise alone. However, two findings emerge as potentially important for future research. First, remotely monitored interventions appear to increase usual gait speed by 0.08 m/s, representing a clinically meaningful improvement in functional mobility for approximately 50% of individuals over the age of 50. Second, the combined intervention seems to improve the gains achieved in other specific physical functions. In fact, compared with physical exercise alone, the combined intervention of cognitive training and physical exercise doubled the gains in fast gait speed (i.e., from 0.08 m/s to 0.15 m/s) and appeared to preserve balance performance better, reducing the small decline of 2.50 s observed in the physical exercise-alone group. Interestingly, although improvements in lower-limb muscular strength were statistically significant in both groups, they remained modest (0.55 s), falling below the minimal detectable improvement threshold (2–3 s). This finding aligns with previous research on home-based physical exercise, showing its lower efficiency in improving lower-limb muscular strength. Also, these small muscular gains are unlikely to account for the observed improvements in functional mobility, suggesting that other mechanisms of progression may be enhanced by cognitive training.

These findings suggest that incorporating cognitive training into a home-based exercise program may support specific components of mobility in adults over 50. Previous studies have reported similar improvements in gross motor functions following low-dose cognitive training programs [45, 46], with training volumes comparable to those performed by participants in the current study (approximately 12 h [19]). These gains in functional mobility have been attributed to the recruitment of central executive processes, targeted during training, that become more involved in motor control with advancing age [8]. In older adults, gait has been linked to compensatory activity in prefrontal regions, reflected by increased activation and reduced hemispheric lateralization in executive-related areas such as the dorsolateral prefrontal cortex [47, 48]. Such a pattern of prefrontal recruitment seems favored by the increased complexity of the motor task [49], such as standing on one leg or walking at their maximal speed.

On the other hand, functional neuroimaging studies suggest that, in older adults, cognitive training might enhance neural efficiency of trained executive control processes (i.e., monitoring, updating, or coordination) by reducing activity in the frontoparietal network and increasing activation in dopaminergic systems [50, 51]. Erickson et al. [52] reported that dual-task training, similar to the computerized tasks used here, improved performance while reducing hemispheric asymmetry and age-related prefrontal overactivation. In the present trial, cognitive training improved executive functioning [19] and increased gains in fast gait speed and one-leg stance, compared to the physical exercise-only group . These tasks, such as fast-paced walking, likely place higher neuromuscular and cardiovascular demands and elicit greater prefrontal cortical activation than walking at a comfortable pace [49, 53, 54]. Hence, these findings may suggest that cognitive training helps maintain or improve mobility in adults after 50 by improving the top-down cognitive control mechanisms involved in motor control.

While some research highlights the potential of computerized cognitive training programs, particularly those targeting attention and executive functions, to improve functional mobility markers such as balance and gait in older adults [15, 45, 55], the literature remains less conclusive regarding the added value of combined training (i.e., cognitive plus physical) compared to physical exercise alone, as observed in the present study [27]. Our results provide valuable insights into the conditions under which cognitive training can lead to significant mobility benefits in older adults, enabling reasonable assumptions about its indications in interventions targeting this population. Several commercial “brain training” programs are being developed and marketed to the public, particularly for older adults, to the extent that dedicated platforms now exist to index cognitive interventions supported by empirical evidence (https://www.cognitivetrainingdata.org/). Within the scientific community, these programs remain controversial in some respects, particularly regarding the extent to which repeated practice, fostering a specific cognitive process, can lead to functionally meaningful generalization to everyday activities such as mobility, decision-making, or multitasking [26, 56]. Regarding its potential for prevention, our study offers a nuanced perspective on how participation in a multidomain intervention may be relevant. Broadly, our results suggest that cognitive training can complement regular physical exercise practice for improving mobility, even when participants performed a substantial amount of physical exercise (i.e., an average of 282 min per week), and may help compensate for the lower effectiveness of physical exercise alone on lower limb functions. However, cognitive training may become particularly relevant when top-down mobility control is required to compensate for increased motor or physiological demands, such as in more complex tasks. Hence, rather than being viewed solely as a general-purpose tool to optimize cognitive functioning across the population regardless of age or physical condition, cognitive training could, for example, be more judiciously integrated into rehabilitation or prevention protocols for older adults, to help individuals when their sensorimotor functions are challenged. Further research should investigate cerebral correlates that support the transfer of cognitive training benefits to motor functions, and the factors, such as training duration, age, or sex, that may moderate this transfer in different populations (e.g., chronic diseases, frail, and mild cognitive impairment).

To fully appreciate the scope of these findings, it is necessary to consider certain study-specific characteristics and their potential impact on the results. First, since the sample size was calculated for the executive function outcome (the published primary outcome), and although this analysis was prespecified in the protocol, the results should be interpreted cautiously. This exploration of mobility outcomes is intended primarily to inform future research on the added value of combining cognitive training with physical exercise in daily-life settings and on self-initiated behaviors. With a sample size of 49 participants per group, the study was adequately powered to detect moderate effect sizes; however, it cannot be excluded that between‑group differences in changes in usual gait speed were too small to be detected. Nevertheless, to some extent, the observation of more robust changes in more complex physical outcomes is, in itself, indicative of the potential relevance of this type of intervention for mobility. Also, the overrepresentation of women in the sample could have contributed to the observed benefits. Previous research suggests that sex-specific physiological adaptations to physical exercise may amplify improvements in both cognitive and physical functioning in females compared to males following physical exercise interventions [33]. However, biological sex does not appear to influence the effects of cognitive training; therefore, any sex-related enhancement of benefits would likely stem from its interaction with physical exercise. Second, because the remote functional assessments used here have not been validated against laboratory-based tests, the values reported, particularly those related to the amplitude of change over time, should be interpreted with caution regarding their clinical significance. Without direct validation, it remains difficult to determine the extent to which remote measurements reflect in‑lab values in terms of absolute accuracy and measurement variability. However, these assessments were based on well-established tools commonly used with older adults and were adapted for supervision via videoconference [39–41]. All assessors were trained to conduct these evaluations online following standardized procedures. The results align with those typically reported in the literature, allowing for scientifically grounded conclusions with a reasonable degree of reliability. Similar caution applies to variables that were necessarily self‑reported, such as height, weight, and perceived exertion. Although these measures may introduce some variability, they were used primarily for descriptive purposes or as covariates and are therefore unlikely to have substantially influenced the interpretation of functional outcomes. Finally, the cognitive training provided included both repeated practice of computerized tasks and the presentation of psychoeducational modules [32]. Based on previous observations of the transfer of benefits from attentional-executive cognitive training to gross motor skills, we structured our interpretations of the benefits observed here around this aspect of the cognitive training. However, it should be noted that, since the provided training was not restricted to this dimension, we cannot exclude improvements in physical performance related to better health behaviors, such as improved sleep or anxiety management, induced by the psycho-educational modules. Hence, while the benefits to executive functioning reported in the clinical trials support the interpretations made here [28], an analysis of exploratory evaluation criteria, such as sleep quality, depressive symptoms, or anxiety, will help strengthen our conclusions. Further research examining the extent to which changes in executive functioning mediate the mobility benefits, particularly by influencing cerebral compensation mechanisms and their evolution with age, could be especially valuable for strengthening our understanding of the underlying mechanisms and improving intervention modalities.

## Conclusions

This secondary analysis of functional mobility outcomes from the COVEPIC trial provides new insights regarding the added value of combining cognitive training with physical exercise in adults aged 50 years and over. The findings suggest that adding cognitive training to home-based physical exercise may enhance specific physical functions, including one-leg balance and fast gait speed. These findings support the interplay between sensorimotor and cognitive functions in older adults and highlight the potential of combined physical and cognitive interventions to prevent age-related mobility decline. Further research is necessary to shed light on the neurocognitive mechanisms underlying the transfer of cognitive training benefits to untrained daily tasks and skills with low degrees of similarity, such as motor skills.

## Supplementary Information

Supplementary Material 1

Supplementary Material 2

## Data Availability

The study dataset is available for the public from the corresponding author upon reasonable request.

## Abbreviations

COVID-19: Coronavirus disease 2019
MoCA: Montreal Cognitive Assessment
NIH: National Institutes of Health
PASE: Physical Activity Scale for the Elderly
PAR-Q+: Physical Activity Readiness Questionnaire
t-MMSE: Mini-Mental Scale Examination telephonic version
