# Supplementary material for: Home-based physical exercice with additional cognitive training for improving mobility in older adults: a secondary analysis of the COVEPIC randomized controlled trial
Source: Eur Rev Aging Phys Act. 2026 May 6;23:25. doi: 10.1186/s11556-026-00415-z (PMC13330402; doi:10.1186/s11556-026-00415-z)
Supplement: Supplementary file 2 — Supplementary Material 2 [file 11556_2026_415_MOESM2_ESM.docx]

*
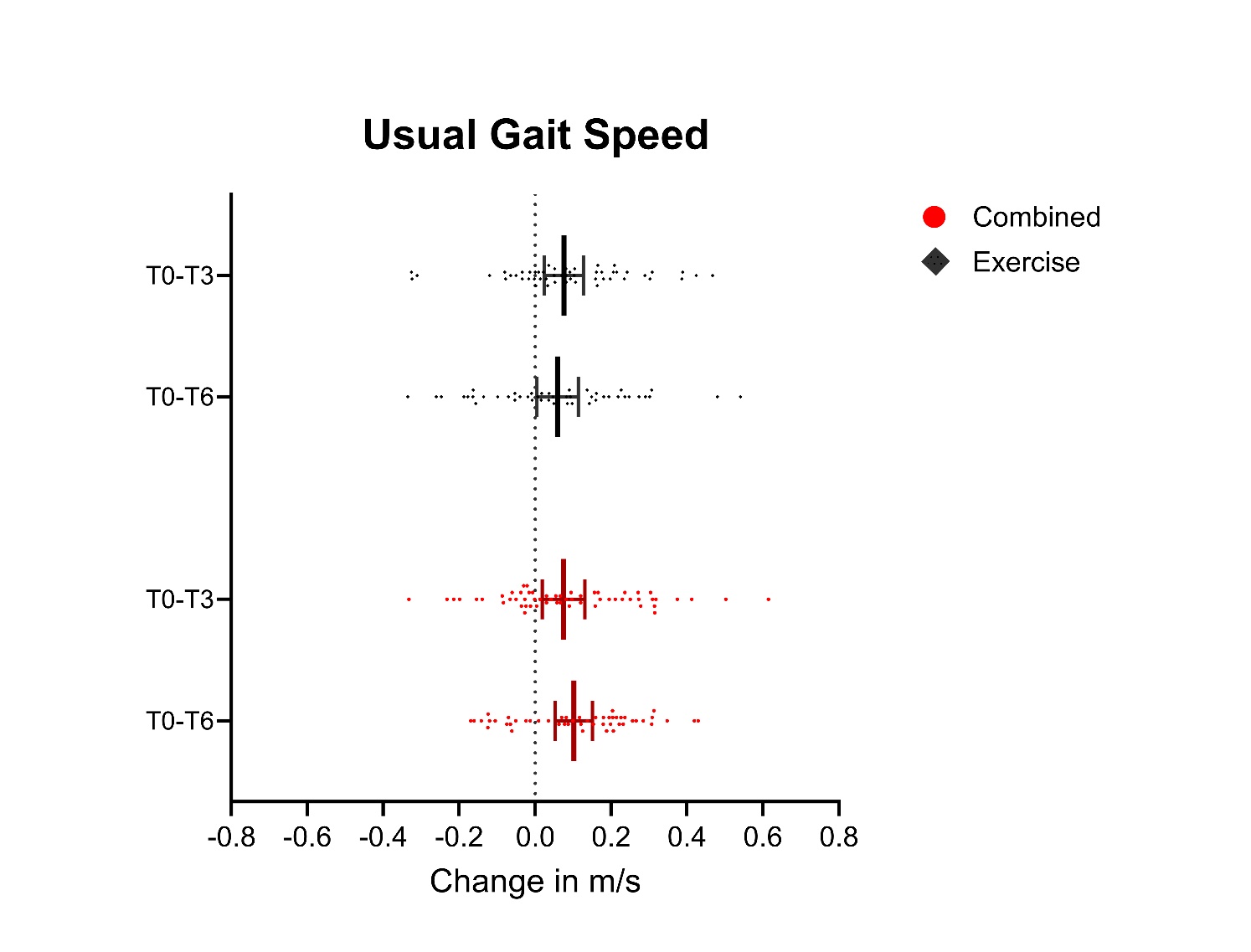
*

**Figure. Usual gait speed changes after three and six months of intervention, according to the study group.**

The figure shows the mean change in usual gait speed between baseline until 3 and 6 months. Error bars represent 95% confidence intervals, and dots indicate individual values.


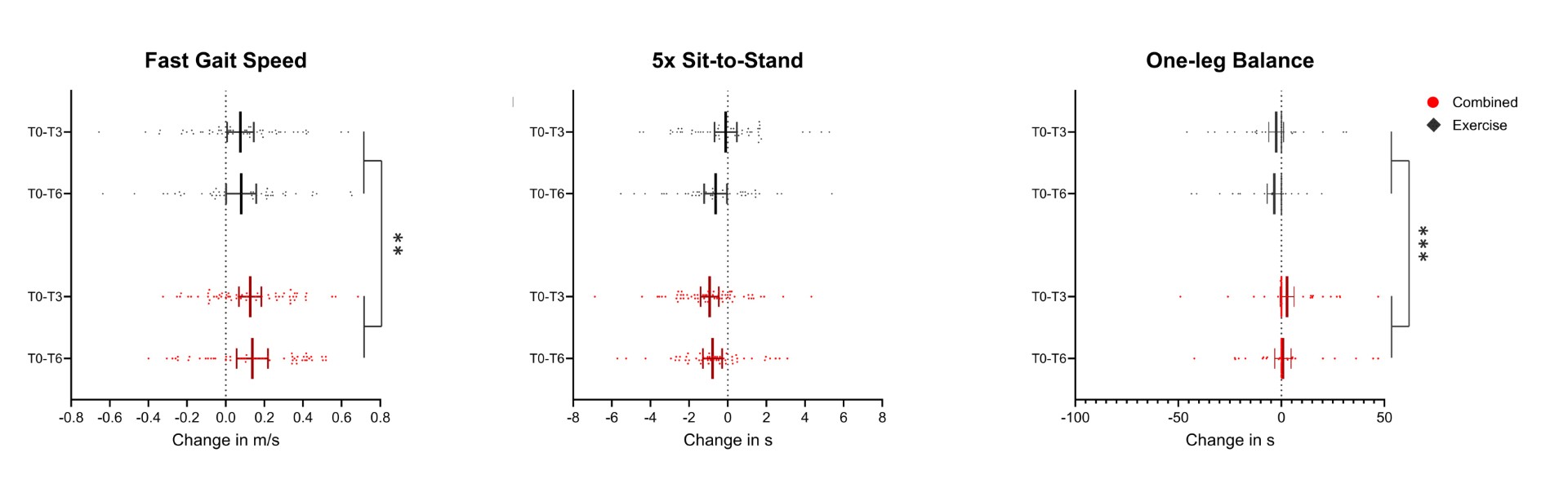


**Figure. Changes in physical functions after three and six months of intervention.**

The figure shows the mean changes in fast gait speed, 5-time sit-to-stand test duration, and one-leg balance test duration from baseline to 3 and 6 months. Error bars represent 95% confidence intervals, and dots indicate individual values. ** p ≤ 0.01; *** p ≤ 0.005.
